# Smoking and drinking among the Gypsy and Traveller communities: A population study in England

**DOI:** 10.1111/add.70330

**Published:** 2026-02-17

**Authors:** Eve Taylor, Harry Tattan‐Birch, Melissa Oldham, Katherine East, Hannah Walsh, Sarah Jackson

**Affiliations:** ^1^ Department of Behavioural Science and Health University College London London UK; ^2^ Department of Primary Care and Public Health, Brighton and Sussex Medical School University of Brighton and University of Sussex Brighton UK; ^3^ Addictions Department, Institute of Psychiatry, Psychology and Neuroscience King's College London London UK

**Keywords:** alcohol, cigarette, drinking, Gypsy, tobacco, traveller

## Abstract

**Background and aims:**

Gypsy and Traveller communities in the United Kingdom (UK) face substantial health challenges. Smoking tobacco and drinking alcohol likely contribute to health disparities, but there is little national data on the prevalence or heaviness of smoking and drinking among these communities. We aimed to estimate the prevalence and heaviness of smoking and drinking among the UK Gypsy and Traveller communities compared with people from other UK ethnic groups.

**Design/setting:**

Observational study using data collected between 2013 and 2025 in a series of monthly cross‐sectional surveys of representative samples of the adult population in England.

**Participants:**

Adults aged 18+, between 2013 and 2025 (total *n* = 226 339; Gypsy or Traveller *n* = 213).

**Measurements:**

Marginal means were derived from regression models and used to estimate the prevalence of current smoking and drinking (both of which includes daily and non‐daily), and the heaviness of smoking (cigarettes per day) and drinking [Alcohol Use Disorders Identification Test‐Consumption (AUDIT‐C) score, units per week and estimated weekly alcohol consumption] by ethnicity; adjusted for age, gender and survey year.

**Findings:**

Current smoking prevalence was markedly higher among Gypsy and Travellers [33.0%, 95% confidence interval (CI) = 26.3–39.8%, *n* = 81] than among the “Other White” ethnic group (18.7%, 95% CI = 18.5–18.9%; *P* < 0.001), and exceeded estimates observed across other ethnic groups (range: 9.4–19.9%; all *P* < 0.001). Among those who smoked, Gypsy and Travellers reported smoking more cigarettes per day (geometric mean = 12.5, 95% CI = 8.9–17.6) than the “Other White” group (geometric mean = 9.0, 95% CI = 8.8–9.1; *P* = 0.59), with other ethnic groups ranging from 6.8–9.7, although not all comparisons reached significance. The proportion reporting any current alcohol consumption was lower among Gypsy and Travellers (61.5%, 95% CI = 53.8–69.2%, *n* = 126) than ‘Other White’ ethnicities (77.1%, 95% CI = 76.8–77.3%; *P* < 0.001). Prevalence of high risk drinking was similar among Gypsy and Travellers (11.3%, 95% CI = 6.7–15.8%) and “Other White” ethnic groups (10.7%, 95% CI = 10.5–10.9%; *P* = 0.808) but exceeded estimates observed across all other ethnic groups (range: 1.3–7.1%; all *P* < 0.05). Prevalence of possible dependence was higher among Gypsy and Travellers (3.5%, 95% CI = 0.4–6.6%) compared with the “Other White” (1.3%, 95% CI = 1.2–1.3%; *P* = 0.027) and all other ethnic groups (range: 0.3–0.8%; all *P* < 0.05).

**Conclusions:**

People from Gypsy and Traveller communities in the United Kingdom appear to be more likely to smoke compared with other UK ethnic groups, and those who smoke and/or drink do so at more harmful levels compared with other UK ethnic groups.

## INTRODUCTION

Within the United Kingdom (UK), ‘Gypsy and Traveller’ are terms used to describe people from a range of distinct ethnicities who are considered to have similar lifestyles and face similar challenges. Terminology to describe people from these ethnic groups varies across Europe, however, in the United Kingdom, Gypsy and Traveller are the official terminology used by the government and by Gypsy and Traveller charities [1, 2]. Gypsy often refers to people who are Romani Gypsy, an ethnic group with origins from Northern India who migrated to the United Kingdom over half a century ago. This community is culturally distinct from people who are Roma, who are people from a Romani background who migrated more recently from central and eastern Europe. The term Traveller primarily refers to Irish Travellers, but can also include New Travellers, Showpeople and Boaters [3]. The 2021 England and Wales Census estimates that 0.1% of the population are Gypsy or Irish Traveller and 0.2% are Roma [4]. This is likely an underestimation because Gypsy and Traveller communities have low engagement with the census, potentially reflecting fears of discrimination, a lack of trust of official reporting, literacy barriers and a nomadic lifestyle meaning some do not have a permanent address [5, 6]. Therefore, although Gypsy and Traveller groups are distinct from each other, they are often reported together and sometimes in combination with Roma communities. For analysis of population surveys, they are also often grouped with other ‘White’ ethnicities as there are limited data available on the individual ethnic groups [7].

Gypsy and Traveller communities face substantial health disparities in the United Kingdom and Europe, with life expectancy estimated to be 5 to 20 years lower than the general population [7, 8]. However, health‐related quality of life differs among these groups, with people who are Roma more likely to report good health (67%) compared to people who are Gypsy or Irish Traveller (60%) [9]. Gypsy and Traveller communities face a disproportionately high burden of respiratory diseases [9, 10], which are commonly associated with tobacco smoking. Regional surveys have documented particularly high smoking rates among this population, for example, 47% (East of England, 2009) and 57% (Hull, 2011) of Gypsy or Irish Travellers were currently smoking [11, 12], more than double the rate reported among the general population during this time period (23% and 22%, respectively) [13]. Within Gypsy and Traveller communities, smoking rates are reportedly similar among men (48%) and women (46%) [11]. More recent data from England's 2022 General Practice (GP) Patient survey estimated that 25% of Gypsy or Irish Travellers were regularly smoking, compared with 11% of people from other White ethnicities [9]. These data, however, may underestimate smoking prevalence, because many Gypsy or Traveller people face barriers when registering with GPs, and would, therefore, not be captured within this data set [14, 15].

Harmful or hazardous alcohol consumption may also contribute to health inequities. Because of a lack of representative data, existing estimates of alcohol consumption among Gypsy and Traveller communities are drawn from unrepresentative surveys. Regional survey data suggests that people who are Gypsy or Traveller are less likely to drink alcohol than then the general population [11, 12, 16]. However, records among the prison population suggest high levels of drug or alcohol problems among the Irish Traveller community (29%) compared to non‐travellers (17%) [17]. These contradictory findings could reflect polarised drinking patterns within Gypsy and Traveller communities (i.e. abstinence or very heavy consumption). There appears to be pronounced gender differences in alcohol use within Gypsy and Traveller communities, as alcohol use is typically associated with masculinity, while female drinking remains uncommon and highly socially regulated [18, 19]. Heavier alcohol use also tends to co‐occur with tobacco use, and drinking at risky levels is more common among those who currently smoke, both in the general population [20] and specifically among Gypsy and Travellers communities [11].

Despite there being evidence that Gypsy and Traveller communities are particularly vulnerable to health outcomes related to smoking and alcohol consumption, the data on health behaviours among this group is very limited to small non‐representative studies, and there are little national data on smoking rates among Gypsy and Traveller communities. To address this gap, we aimed to answer the following research questions:

1. How does the prevalence of (a) current tobacco smoking; (b) any alcohol consumption; and (c) risky/harmful/possible dependent drinking among people who are Gypsy or Travellers compare with that of other ethnic groups?
2. Among those who smoke, is there a difference in the average number of cigarettes smoked per day between people who are Gypsy or Traveller and those from other ethnic groups?
3. Among those who drink alcohol, is there a difference in average Alcohol Use Disorders Identification Test‐Consumption (AUDIT‐C) score and estimated units of alcohol consumed per week, between people who are Gypsy or Travellers and those from other ethnic groups?
4. To what extent do smoking and risky drinking co‐occur among people who are Gypsy or Traveller and those from other ethnic groups?

## METHODS

### Pre‐registration

The study protocol, research questions and analysis plan were pre‐registered on Open Science Framework (https://osf.io/7j6mp/). In addition to our pre‐registered analyses, we also conducted exploratory analyses examining differences in harmful and possible dependent drinking, Estimator of Weekly Alcohol Consumption scale (EWAC) score and interactions between smoking and drinking.

### Design

Data were drawn from the Smoking and Alcohol Toolkit Studies, an ongoing monthly cross‐sectional survey of adults (≥16 years) in England [21, 22]. The study uses a hybrid of random probability and simple quota sampling to select a new sample of approximately 1700 adults in England each month. A comprehensive question on ethnicity was added in January 2013, which captures people who are Gypsy or Traveller (but not Roma). Measures on alcohol consumption were added in March 2014. Data were originally collected via face‐to‐face interviews, but data collection switched to telephone interviews from April 2020 onward. The two modes show good comparability on key socio‐demographic and smoking indices [23]. Because interviewers are not given a fixed list of addresses to approach, an accurate response rate cannot be calculated for this survey design. All participants provided oral consent.

### Participants

A total of 238 416 adult (18+ years) participants were surveyed in England between January 2013 and February 2025. Data on ethnicity were not collected between April and August 2020, so data for these waves were excluded (*n* = 8459). A total of 226 339 (98.6%) provided complete data on the covariates of interest (age, gender) for smoking analyses (January 2013–February 2025), and 206 610 (98.5%) for alcohol analyses (March 2014–February 2025).

### Measures

See pre‐registration for full details of survey measures (https://osf.io/7j6mp/).

Smoking status was assessed by asking participants which of the following statements best applied to them (a) I smoke cigarettes (including hand‐rolled) every day; (b) I smoke cigarettes (including hand‐rolled), but not every day; (c) I do not smoke cigarettes at all, but I do smoke tobacco of some kind (e.g. pipe, cigar or shisha); (d) I have stopped smoking completely in the last year; (e) I stopped smoking completely more than a year ago; (f) I have never been a smoker (i.e. smoked for a year or more). Responses were coded ‘current smoking (a–c)’ and ‘other (d–f)’. Those who did not know which smoking status descriptor best applied to them were excluded from smoking analysis (*n* = 709, 0.31%). Smoking measures are validated against national surveys and cigarette sales data [22, 24].

Cigarettes per day was derived among those who currently smoked cigarettes. Participants were asked: ‘How many cigarettes do you usually smoke?’ They could respond using figures for a day, a week or (in some waves) a month. Participants who replied ‘do not know’ were encouraged to provide their best estimates. Responses were converted to cigarettes consumed per day (i.e. either the number of cigarettes per day, or the number per week divided by 7 or per month divided by 30). Responses above 80 cigarettes per day were considered implausible and were, therefore, replaced with a value of 80. Cigarettes per day is a validated measure of the heaviness of smoking [25].

Alcohol outcomes were derived from the AUDIT‐C questionnaire, a validated measure of alcohol consumption [26]. Current drinking (yes/no) was derived from the first AUDIT‐C question. Heaviness of drinking was derived from all three AUDIT‐C questions and was analysed among all participants as three binary variables indicating risky drinking (AUDIT‐C ≥5), harmful drinking (AUDIT‐C ≥8) and possible dependence (AUDIT‐C ≥11).

Among those who reported current drinking, units of alcohol consumed per week were calculated based on responses to questions 1 and 2 of the AUDIT‐C​ [17]​. Those who responded do not know (*n* = 1834, 0.89%) or refused (*n* = 735, 0.35%) to the AUDIT‐C questions were excluded from alcohol analysis. Among those who drank, the EWAC scores were also calculated (audit1 × audit2 + audit3) [27]. The EWAC incorporates responses to question 3 of the AUDIT‐C (frequency of more than 6 units consumed in a single occasion), therefore, is more sensitive to binge drinking than standard units per week calculations [27].

Ethnicity was categorised as ‘Gypsy or Traveller’, ‘Other White’ (including ‘White English, Welsh, Scottish, Northern Irish or British’, ‘White Irish’, ‘White other’), ‘Asian, British Asian’, ‘Black, Black British’, ‘Mixed or multiple ethnicities’, ‘Arab’, or ‘Other Ethnicity (including don't know and refused)’.

Covariates included survey year (continuous), age (continuous) and gender (man, woman, in another way). For regression models, those identifying in another way were excluded because of small sample sizes (*n* = 737, 0.3%). In addition, we included a binary variable to account for changes in survey methodology around this time (from in‐person to phone interviews) resulting from the coronavirus disease 2019 (COVID‐19) pandemic ​ [21].

### Analyses

Data were pooled across all available years (smoking 2013–2025; alcohol 2014–2025).

Cigarettes per day, alcohol units per week and EWAC score were log transformed to account for skewness of data.

We used descriptive analyses to report participants' socio‐demographic characteristics (*n* and %), the prevalence and heaviness of current tobacco smoking and the prevalence and heaviness of alcohol consumption.

Among all participants, logistic regression examined the association between ethnicity and (a) current smoking; (b) current drinking; (c) risky drinking; (d) harmful drinking; and (e) possible dependent drinking. Predictive margins from these models were derived to estimate the marginal adjusted prevalence for each ethnicity, as well as relative risk ratios and 95% CIs. Analyses were repeated stratified by gender.

Among those who reported current cigarette smoking, linear regressions investigated the associations between ethnicity and daily cigarette consumption. Among those who reported current drinking, linear regressions investigated the associations between ethnicity and (a) weekly units of alcohol consumed and (b) EWAC score. Predictive margins for these models were derived to obtain the adjusted geometric mean (GM) for each ethnic group and adjusted GM ratios and 95% CIs. Analyses were repeated stratified by gender.

Additional to pre‐registration, to explore associations between smoking and risky drinking, logistic regression examined the association between ethnicity and risky drinking, including an interaction term for smoking. Predictive margins from these models were derived to estimate the marginal adjusted prevalence for each ethnicity. Adjusted prevalence and adjusted GMs were plotted by ethnicity for each outcome.

All regression analyses were weighted to match the English population profile [19], and adjusted for age, gender, survey year and the onset of the COVID‐19 pandemic. Socio‐economic status was not included as a covariate because it is a mediator of the association between ethnicity and smoking/alcohol outcomes, and adjusting for it would remove variance attributable to structural inequalities.

## RESULTS

Overall, 0.1% (213) of the sample were Gypsy or Traveller, 84.3% were from other White ethnicities, 2.0% were from mixed or multiple ethnicities, 7.4% were Asian/British Asian, 4.3% were Black/Black British, 0.3% were Arab and 1.6% identified as a different ethnicity. Characteristics of this analytical sample are provided in Table 1.

**TABLE 1:** Participant characteristics.

	Weighted	Unweighted		
	All participants	All participants (*n* = 226 339)	Participants who currently smoke (*n* = 38 473)^a^	Participants who currently drink (*n* = 146 022)^b^
	%	% (*n*)	% (*n*)	% (*n*)
Ethnicity				
Gypsy or Traveller	0.1	0.1 (213)	0.2 (81)	0.1 (126)
Other White	85.5	84.3 (190 841)	87 (33 479)	90.8 (132 606)
Mixed/multiple ethnicity	1.9	2.0 (4517)	2.7 (1036)	2.0 (2926)
Asian/British Asian	6.8	7.4 (16 708)	5.2 (1996)	2.6 (3827)
Black/Black British	3.9	4.3 (9747)	2.9 (1096)	3.1 (4519)
Arab	0.3	0.3 (743)	0.4 (161)	0.1 (134)
Other/do not know/refused	1.5	1.6 (3570)	1.6 (624)	1.3 (1884)
Gender				
Men	48.9	50.1 (113 450)	53.6 (20 635)	52.4 (76 500)
Women	50.8	49.6 (112 152)	45.9 (17 639)	47.3 (69 012)
In another way^c^	0.3	0.3 (737)	0.5 (199)	0.4 (510)
Socio‐economic status				
ABC1	55.5	60.7 (137 369)	45.3 (17413)	66.7 (97 415)
C2DE	44.5	39.3 (88 970)	54.7 (21060)	33.3 (48 607)
Age	M = 48.0 (SD=18.6)	M = 49.42 (SD = 19.1)	M = 43.4 (SD = 17.2)	M = 50.0 (SD = 18.7)

^a^ Including those who were smoking non‐cigarette tobacco (*n* = 2083, 0.92%). Excluding those who did not currently smoke (*n* = 187 157, 82.69%) or did not know if they had smoked (*n* = 709, 0.31%).

^b^ Excluding those who did not currently drink (28.65%), did not know if they drink (0.24%) or refused the question (0.43%).

^c^ Participants who identified ‘In another way’ were excluded from regression analysis because of small cell counts.

### Smoking

Among people who were Gypsy or Traveller, 34.1% smoked cigarettes daily, 2.2% smoked non‐daily and 3.0% exclusively smoked non‐cigarette tobacco. Just under half had never regularly smoked (46.0%), while 14.7% had smoked regularly in the past. See Figure S1 for comparisons with other ethnicities.

**FIGURE 1:**
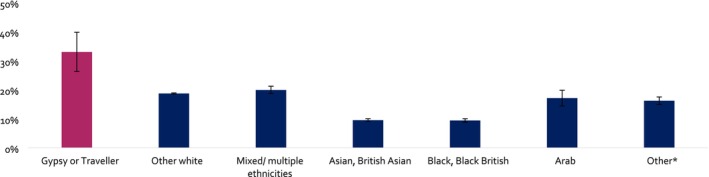
Prevalence of current smoking by ethnicity: pooled 2013–2025.

People who were Gypsy or Traveller had higher prevalence of current smoking compared with any other ethnic group (adjusted prevalence = 33.0% Gypsy or Traveller; 9.4%–19.9% other ethnic groups) (Figure 1, Table 2). Among those who currently smoked cigarettes, Gypsy or Travellers had the highest estimated daily cigarette consumption, with an adjusted GM of 12.5 cigarettes per day. In contrast, the estimated consumption for other ethnic groups ranged from GM = 6.7 to 9.7 cigarettes per day. However corresponding CIs for comparisons with other White, Arab and other ethnic groups included the possibility of no difference (Figure S2, Table 2). Similar patterns were found when analyses were repeated, stratified by gender (Table 2).

**TABLE 2:** Smoking among in adults from Gypsy or Traveller communities compared with other ethnic groups; January 2013–February 2025 (*n* = 226 339).

Current smoking^a^	All participants			Men			Women		
	Adjusted prevalence (95% CI)	ARR (95% CI)	Sig	Adjusted prevalence (95% CI)	ARR (95% CI)	Sig	Adjusted prevalence (95% CI)	ARR (95% CI)	Sig
Gypsy or Traveller	33.0% (26.3–39.8)	1.00	Ref	35.9 (26.6–45.3)	1.00	Ref	30.0 (20.3–39.8)	1.00	Ref
Other White	18.7% (18.5–18.9)	0.56 (0.45–0.68)	**<0.001**	20.0 (19.7–20.3)	0.56 (0.41–0.70)	**<0.001**	17.3 (17.1–17.6)	0.58 (0.39–0.77)	0.**003**
Mixed/multiple ethnicity	19.9% (18.7–21.2)	0.60 (0.47–0.73)	**<0.001**	21.2 (19.3–23.0)	0.59 (0.43–0.75)	0.**001**	18.7 (17.1–20.3)	0.62 (0.41–0.83)	0.**010**
Asian/British Asian	9.5% (9.1–10.0)	0.29 (0.23–0.35)	**<0.001**	14.2 (13.5–15.0)	0.40 (0.29–0.50)	**<0.001**	3.8 (3.4–4.3)	0.13 (0.08–0.17)	**<0.001**
Black/Black British	9.4% (8.8–10.0)	0.29 (0.22–0.35)	**<0.001**	11.7 (10.8–12.7)	0.33 (0.24–0.42)	**<0.001**	7.3 (6.6–8.0)	0.24 (0.16–0.32)	**<0.001**
Arab	17.1% (14.4–19.8)	0.52 (0.38–0.65)	**<0.001**	23.3 (19.3–27.2)	0.65 (0.45–0.85)	**<0.001**	8.3 (5.1–11.5)	0.28 (0.14–0.41)	**<0.001**
Other^c^	16.2% (14.9–17.5)	0.49 (0.38–0.60)	**<0.001**	19.4 (17.5–21.2)	0.54 (0.39–0.69)	**<0.001**	12.8 (11.0–14.5)	0.42 (0.28–0.57)	**<0.001**
Cigarettes per day^**b**^	AGM (95% CI)	AGMR (95% CI)		AGM (95% CI)	AGMR (95% CI)		AGM (95% CI)	AGMR (95% CI)	
Gypsy or Traveller	12.5 (8.9–17.6)	1.00	Ref	14.7 (9.1–23.7)	1.00	Ref	10.0 (6.3–15.8)	1.00	Ref
Other White	9.0 (8.8–9.1)	0.72 (0.51–1.01)	0.059	9.7 (9.5–9.9)	0.66 (0.41–1.07)	0.091	8.1 (8.0–8.3)	0.81 (0.52–1.29)	0.380
Mixed/multiple ethnicity	7.1 (6.4–7.7)	0.56 (0.40–0.81)	0.**002**	7.8 (6.9–9.0)	0.53 (0.32–0.88)	0.**013**	6.3 (5.5–7.1)	0.63 (0.39–1.01)	0.053
Asian/British Asian	6.8 (6.3–7.2)	0.54 (0.38–0.77)	0.**001**	7.3 (6.7–7.8)	0.49 (0.30–0.80)	0.**004**	6.8 (5.5–8.3)	0.68 (0.41–1.12)	0.129
Black/Black British	6.8 (6.1–7.4)	0.54 (0.38–0.77)	0.**001**	7.1 (6.2–8.1)	0.48 (0.29–0.79)	0.**004**	6.5 (5.6–7.5)	0.65 (0.40–1.04)	0.075
Arab	9.7 (7.9–12.0)	0.78 (0.52–1.17)	0.225	11.0 (9.0–13.4)	0.75 (0.45–1.25)	0.269	8.0 (3.6–17.4)	0.80 (0.32–1.97)	0.620
Other^c^	8.8 (7.9–9.9)	0.71 (0.49–1.01)	0.059	9.8 (8.5–11.3)	0.67 (0.41–1.10)	0.112	7.7 (6.4–9.3)	0.77 (0.47–1.26)	0.304

*Notes:* Analysis is adjusted for age, gender, survey year and the onset of the COVID‐19 pandemic. Adjusted prevalence‐average predictive margins derived from the logistic regression were used to estimate the prevalence of smoking for each ethnicity adjusted for age, gender, survey year and COVID. Bold denotes a *P* value of <0.05.

Abbreviations: AGM, adjusted geometric mean; AGMR, adjusted geometric mean ratio; ARR, adjusted relative risk, calculated based on the adjusted prevalence; COVID‐19, coronavirus disease 2019; Sig, statistical significance. Significance was derived from the logistic regression model.

^a^ Excludes those who did not know which smoking status descriptor best applied to them (*n* = 709, 0.31%).

^b^ Among people who currently smoke cigarettes (*n* = 36 390). Excludes those who were smoking non‐cigarette tobacco (*n* = 6084, 2.69%).

^c^ ‘Other’ includes those who reported they were an other ethnicity, do not know or refused.

### Drinking

**FIGURE 2:**
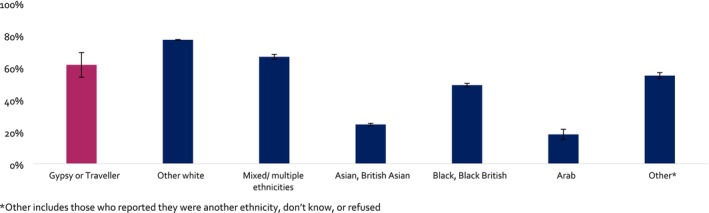
Prevalence of current alcohol drinking by ethnicity: pooled 2014–2025.

People who were Gypsy or Traveller had lower prevalence of current drinking compared with people from other White ethnicities (Gypsy or Traveller adjusted prevalence = 61.5%, other White = 77.1%), but higher prevalence than those who were Asian, British Asian (24.4%), Black, Black British (48.9%) or Arab (18.2%) (Figure 2, Table 3).

**TABLE 3:** Drinking among adults from Gypsy or Traveller communities compared with other ethnic groups; January 2014–February 2025 (*n* = 206 610).

Currently drinking^a^	All participants			Men			Women		
	Adjusted prevalence (95% CI)	ARR (95% CI)	Sig	Adjusted prevalence (95% CI)	ARR (95% CI)	Sig	Adjusted prevalence (95% CI)	ARR (95% CI)	Sig
Gypsy or Traveller	61.5 (53.8–69.2)	1.00	Ref	64.2 (54.3–74.2)	1.00	Ref	61.0 (49.5–72.6)	1.00	Ref
Other White	77.1 (76.8–77.3)	1.25 (1.10–1.41)	**<0.001**	81.4 (81.1–81.6)	1.27 (1.07–1.46)	**<0.001**	72.9 (72.6–73.3)	1.19 (0.97–1.42)	0.**029**
Mixed/multiple ethnicity	66.5 (65.0–68.1)	1.08 (0.94–1.22)	0.194	69.5 (67.2–71.8)	1.08 (0.91–1.25)	0.295	63.6 (61.4–65.8)	1.04 (0.84–1.24)	0.666
Asian/British Asian	24.4 (23.6–25.1)	0.40 (0.35–0.45)	**<0.001**	28.0 (27.0–29.1)	0.44 (0.37–0.51)	**<0.001**	21.4 (20.3–22.5)	0.35 (0.28–0.42)	**<0.001**
Black/Black British	48.9 (47.8–50.0)	0.80 (0.69–0.90)	0.**002**	52.6 (50.9–54.3)	0.82 (0.69–0.95)	0.**031**	45.4 (43.8–46.9)	0.74 (0.60–0.89)	0.**011**
Arab	18.2 (15.1–21.4)	0.30 (0.23–0.36)	**<0.001**	20.8 (16.3–25.3)	0.32 (0.24–0.41)	**<0.001**	17.0 (12.3–21.7)	0.28 (0.18–0.37)	**<0.001**
Other^c^	54.8 (53.0–56.7)	0.89 (0.78–1.01)	0.107	58.8 (56.3–61.3)	0.92 (0.77–1.06)	0.313	51.8 (49.1–54.5)	0.85 (0.68–1.02)	0.137
Risky drinking (AUDIT‐C ≥5)^b^									
Gypsy or Traveller	26.5 (19.9–33.1)	1.00	Ref	33.5 (23.7–43.4)	1.00	Ref	20.9 (11.9–29.9)	1.00	Ref
Other White	32.6 (32.3–32.8)	1.23 (0.92–1.53)	0.090	42.3 (42.0–42.7)	1.26 (0.89–1.63)	0.099	23.2 (22.9–23.5)	1.11 (0.63–1.59)	0.628
Mixed/multiple ethnicity	22.8 (21.5–24.1)	0.86 (0.64–1.08)	0.260	28.0 (25.8–30.1)	0.83 (0.58–1.09)	0.259	17.4 (15.9–18.9)	0.83 (0.47–1.20)	0.424
Asian/British Asian	5.4 (5.0–5.7)	0.20 (0.15–0.25)	**<0.001**	7.7 (7.1–8.4)	0.23 (0.16–0.30)	**<0.001**	3.2 (2.8–3.6)	0.15 (0.08–0.22)	**<0.001**
Black/Black British	9.2 (8.6–9.8)	0.35 (0.26–0.44)	**<0.001**	12.0 (11.0–13.1)	0.36 (0.25–0.47)	**<0.001**	6.4 (5.8–7.1)	0.31 (0.17–0.44)	**<0.001**
Arab	5.1 (3.5–6.7)	0.19 (0.12–0.27)	**<0.001**	6.0 (3.5–8.5)	0.18 (0.09–0.27)	**<0.001**	5.1 (2.8–7.4)	0.24 (0.09–0.39)	**<0.001**
Other^c^	14.7 (13.5–16.0)	0.55 (0.41–0.70)	**<0.001**	21.1 (19.0–23.1)	0.63 (0.43–0.82)	0.**007**	8.7 (7.2–10.1)	0.41 (0.22–0.61)	0.**001**
High risk drinking (AUDIT‐C ≥8)^b^									
Gypsy or Traveller	11.3 (6.7–15.8)	1.00	Ref	16.0 (8.4–23.7)	1.00	Ref	7.2 (2.0–12.3)	1.00	Ref
Other White	10.7 (10.5–10.9)	0.95 (0.57–1.34)	0.808	15.8 (15.5–16.1)	0.98 (0.51–1.45)	0.947	5.8 (5.7–6.0)	0.81 (0.23–1.40)	0.579
Mixed/multiple ethnicity	7.1 (6.3–7.9)	0.63 (0.37–0.89)	0.**035**	9.5 (8.1–10.8)	0.59 (0.30–0.88)	0.**045**	4.6 (3.8–5.4)	0.64 (0.17–1.11)	0.245
Asian/British Asian	1.5 (1.3–1.7)	0.13 (0.07–0.18)	**<0.001**	2.4 (2.0–2.7)	0.1 5 (0.07–0.22)	**<0.001**	0.6 (0.4–0.7)	0.08 (0.02–0.14)	**<0.001**
Black/Black British	2.1 (1.8–2.4)	0.19 (0.11–0.27)	**<0.001**	2.9 (2.4–3.5)	0.18 (0.09–0.28)	**<0.001**	1.3 (1.0–1.6)	0.18 (0.04–0.32)	**<0.001**
Arab	1.3 (0.5–2.0)	0.11 (0.03–0.20)	**<0.001**	1.7 (0.5–2.9)	0.11 (0.02–0.20)	**<0.001**	1.0 (0.0–2.2)	0.14 (0.00–0.33)	0.**004**
Other^c^	4.8 (4.1–5.6)	0.43 (0.24–0.62)	**<0.001**	8.0 (6.7–9.4)	0.5 (0.25–0.76)	0.**011**	1.7 (1.0–2.4)	0.23 (0.04–0.43)	0.**001**
Possible dependent drinking (AUDIT‐C ≥11)^b^									
Gypsy or Traveller	3.5 (0.4–6.6)	1.00	Ref	5.0 (0.0–10.5)	1.00	Ref	2.3 (0.0–5.6)	1.00	Ref
Other White	1.3 (1.2–1.3)	0.36 (0.04–0.68)	0.**027**	2.1 (2.0–2.2)	0.41 (0.00–0.86)	0.117	0.5 (0.5–0.6)	0.22 (0.00–0.53)	0.**039**
Mixed/multiple ethnicity	0.9 (0.6–1.1)	0.24 (0.01–0.47)	0.**004**	1.5 (1.0–2.0)	0.29 (0.00–0.63)	0.**041**	0.3 (0.1–0.5)	0.11 (0.00–0.30)	0.**010**
Asian/British Asian	0.3 (0.2–0.3)	0.07 (0.01–0.14)	**<0.001**	0.4 (0.3–0.6)	0.08 (0.00–0.18)	**<0.001**	0.1 (0.0–0.2)	0.04 (0.00–0.11)	0.**001**
Black/Black British	0.3 (0.2–0.4)	0.08 (0.01–0.16)	**<0.001**	0.5 (0.2–0.7)	0.09 (0.00–0.20)	**<0.001**	0.1 (0.0–0.2)	0.04 (0.00–0.12)	**<0.001**
Arab	0.3 (0.0–0.7)	0.07 (0.00–0.20)	0.**003**	0.4 (0.0–1.1)	0.07 (0.00–0.23)	0.**021**	0.2 (0.0–0.6)	0.0 9 (0.00–0.29)	0.**047**
Other^c^	0.8 (0.5–1.1)	0.22 (0.01–0.43)	0.**003**	1.4 (0.9–2.0)	0.28 (0.00–0.60)	0.**034**	0.2 (0.0–0.4)	0.07 (0.00–0.21)	0.**006**

*Notes:* Analysis is adjusted for age, gender, survey year and the onset of the COVID‐19 pandemic. Bold denotes a *P* value of <0.05.

Abbreviations: ARR, adjusted relative risk; AUDIT‐C, Alcohol Use Disorder Identification Test‐Consumption; Sig, statistical significance. Significance was derived from the logistic regression model.

^a^ Excludes those who did not know if they drank (*n* = 498, 0.24%) or refused (*n* = 898, 0.43%).

^b^ Excludes those who responded do not know or refused to any Audit question (*n* = 3403, 1.6%).

^c^ ‘Other’ includes those who reported they were another ethnicity, do not know or refused.

**FIGURE 3:**
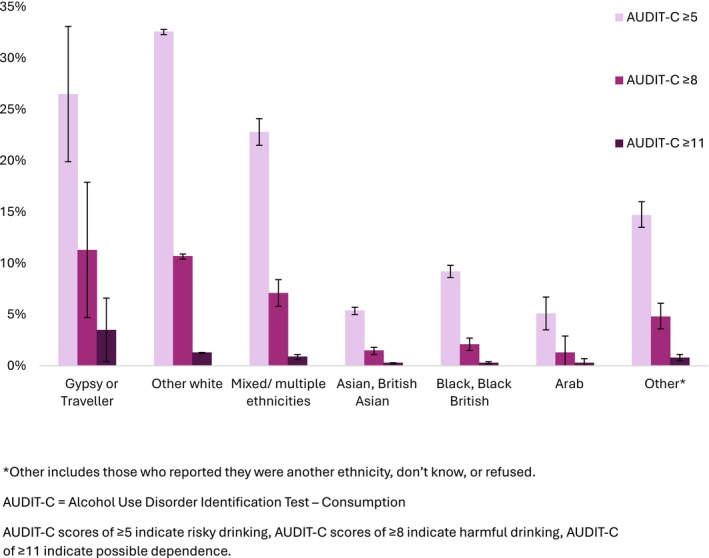
Audit‐C scores by ethnicity: pooled 2014–2025. Audit‐C, Alcohol Use Disorder Identification Test‐Consumption.

Prevalence of risky drinking (AUDIT‐C ≥5) was lower among Gypsy or Travellers (26.5%) compared with other White ethnicities (32.6%) and higher than other ethnic groups (5.1%–22.8%). However, heavy drinking (AUDIT‐C ≥8), and possible dependent drinking (AUDIT‐C ≥11) were more prevent among Gypsy or Travellers (heavy 11.3%, dependent 3.5%) than other White ethnicities (heavy 10.7%, dependent 1.4%) and other ethnic minorities (heavy 1.3%–7.1%, dependent 0.3%–0.9%) (Figure 3, Table 3). Cell counts for heavy and possible dependent drinking were small, so findings should be interpreted with caution (Table 3).

Among those who currently drink alcohol (*n* = 144 487), Gypsy or Travellers drank a similar number of units per week (adjusted GM = 3.5), compared to other White ethnicities (GM = 3.4) and people from mixed/multiple ethnicities (M = 2.66), but drank more units per week than all other ethnicities (GM = 1.41–2.21) (Figure S3a, Table S1). Those who were Gypsy or Travellers who drank also had similar EWAC scores as people who were from other White ethnicities (GM = 8.2 vs. 7.6), but higher scores than people from all other ethnic groups (GM = 6.7–5.2) (Figure S3b, Table S1).

When analyses were stratified by gender, the differences in drinking between Gypsy or Traveller individuals and those from other ethnic groups were comparable for men and for women. The prevalence of current drinking among Gypsy or Traveller men and women was similar (men = 64.22%; women = 61.04%), but risky (33.54%; 20.91%), heavy (16.04%; 7.15%), possible dependent drinking (5.03%; 2.31%) and units per week (GM = 4.72; GM = 2.60) were higher among Gypsy or Traveller men than women (Table 3, Table S1).

### Smoking and drinking interactions

When prevalence of risky drinking (AUDIT‐C ≥5) was compared by smoking status, risky drinking was more prevalent among people who were Gypsy or Traveller who smoked [adjusted prevalence = 33.4% (95% CI = 22.0%–44.9%)] compared with those who did not smoke [21.8% (13.8%–29.7%)]. However, the wide and overlapping CIs reflect small sample sizes and include the possibility of no difference (*P* = 0.101) (Figure S4).

## DISCUSSION

The findings of this study reveal substantial disparities in smoking and alcohol consumption patterns among Gypsy and Traveller communities compared with other ethnic groups in the United Kingdom. Current smoking prevalence and average daily cigarette consumption were substantially greater among Gypsy and Travellers than any other ethnicity. Current alcohol consumption was lower among Gypsy and Travellers than other White ethnicities, but the prevalence of heavy drinking and possible dependent drinking were greatest among Gypsy and Traveller communities compared with all other ethnic groups.

High levels of smoking are consistent with previous research among Gypsy and Traveller communities [9, 11]. These communities face high levels of discrimination, which can lead to stress anxiety and the use of smoking as a coping mechanism [4, 28]. Additionally, structural discrimination in the workforce means that many Gypsy and Travellers work in manual occupations or are economically inactive because of long term disability, both of which are associated with higher smoking prevalence [29, 30, 31]. Another factor that may contribute to the high rates of smoking among these communities is lack of access or knowledge of smoking cessation support. Additionally, qualitative research describes how these communities' health beliefs, such as low expectations of health and a need for self‐reliance, may reduce engagement with support services [32]. Previous studies have found that although the majority of Gypsy or Irish Travellers show a willingness to quit smoking (61%), few have used health services, prescription medication or alternative nicotine products to help with cessation (31%) [11]. Using evidence‐based support for a quit attempt, such as behavioural support, nicotine replacement therapy, prescription pharmaceutical or e‐cigarettes can improve the likelihood of quitting smoking [33, 34].

Gypsy and Travellers communities reported lower overall prevalence of alcohol consumption than other White ethnic groups, consistent with previous research [11, 12, 16]. However, Gypsy and Traveller communities had disproportionately higher rates of heavy and possible dependent drinking, particularly among men. Gender discrepancies in drinking could be related to cultural norms in Gypsy and Traveller communities [18]. Drinking among men is tolerated and seen as part of networking and finding work through local pubs. Whereas, drinking among women, particularly those who are younger and unmarried, is discouraged [18]. Dependent drinking, however, is highly stigmatised within the Gypsy and Traveller communities and seen as shameful [35]. Therefore, these groups may be less likely to seek external help [18], and when they do seek help, this is typically at a very late stage when dependence has already developed [19], possibly explaining the high frequency of heavy and dependent drinking.

Findings highlight the need for greater support services for Gypsies and Travellers who smoke and/or drink. These communities report high levels of discrimination within society in general, and specifically within health care services, leading to a disengagement with healthcare systems [9, 36]. High levels of stigma toward heavy drinking in these communities may further compound this disengagement. The development of trust using culturally competent outreach between Gypsies and Travellers and healthcare professionals is key to increase awareness of health risks and the use of tobacco and alcohol services [37]. Evidence for this includes programmes where community leaders worked with health care professionals to provide smoking cessation training opportunities to peer mentors, so that support could be provided by community members [38]. Trust and respect can also be developed when Gypsy and Traveller communities are consulted on how they would like services to support them. Cultural awareness training among staff has also been reported as a strategy to improve access, but with the caution that this can risk reinforcing stereotypes [39].

Specific focus may also be needed to improve women's health. Almost a third of Gypsy or Traveller women reported smoking, and previous research has reported that a high number of Gypsy or Traveller women continue to smoke throughout pregnancy [40]. Gypsy and Traveller women face higher risks of maternal health complications and stillbirth [10, 41, 42]. The reasons for poor maternal outcomes are multi‐factorial, including discrimination from healthcare professionals, a lack of awareness or understanding of healthcare services, higher rates of birth complications and also issues surrounding dietary supplements and cigarette smoking [41, 42]. Smoking cessation programmes, targeting pregnant Gypsy and Traveller women are required to improve maternal and infant outcomes. The inclusion of these communities within the UK smoke‐free pregnancy incentive scheme, for example, should be measured.

Services should, however, not only focus on specialist outreach, but should also make mainstream services more flexible and accessible to the Gypsy and Traveller communities, to ensure that they do not become further isolated from healthcare provision [37]. For example, people who are Gypsies or Travellers are less aware of alcohol treatment services and perceive health care services as hard to access [18]. Therefore, assistance is needed in helping people understand healthcare pathways, access and referral routes and recognize the potential impact of literacy and language barriers, because these can mean that people struggle to complete forms and communicate their healthcare needs [43]. There have been many outreach and engagement schemes at local trust or council level, however, national strategy and funding could better improve Gypsy and Traveller access to tobacco and alcohol service provision, given the high prevalence identified in the current study [37].

Our research has several limitations. Our measure of ethnicity grouped people who are Gypsy and Traveller into one category, which may mask possible variations in smoking and drinking behaviours among these communities. Moreover, as some people from the Gypsy and Traveller communities do not have a permanent address, previous research has reported that it can be difficult to accurately sample these communities, leading to underestimation of smoking levels [44]. These groups may have been under‐represented when our data collection was using in person household survey methods, however, our sampling methods moved to telephone interviews in 2020, therefore, we were able to reach anyone with a mobile phone, potentially allowing for greater inclusion of Gypsy and Travellers without permanent addresses. The Gypsy and Traveller communities are also known to be wary of data collection about their community, as is evidenced by the low participation in the National Census [2, 5]. Therefore, the people who participated may not be wholly representative of Gypsy and Traveller communities. Additionally, small sample sizes for people who are Gypsy or Traveller mean estimates are less precise than those for larger groups. Because of our data collection methods we were unable to estimate smoking and drinking rates among the Roma community. Although, Gypsy, Traveller and Roma communities are distinct, they all face similar health inequalities and discriminations [7], and previous surveys among Roma have indicated a similar smoking prevalence to Gypsy and Travellers [9]. Therefore, future research is needed among the Roma community, and outreach programs must ensure that support if provided to the three communities. Additional research could also explore if there are similar differences between ethnicities in the use of e‐cigarette and other novel nicotine products.

Overall, this study suggests that Gypsy and Traveller communities who drink and/or smoke do so at more harmful levels than other ethnic groups. This likely contributes to the significant health inequalities experienced by this group [7, 8]. Because people who are Gypsy or Travellers generally access healthcare services less often and experience stigma from healthcare professionals, nationally funded programs of culturally competent outreach and improved service accessibility are needed among this community to engage them in tobacco and alcohol treatment services, improve prevention and reduce health inequalities.

## AUTHOR CONTRIBUTIONS

**Eve Taylor:** Conceptualization; writing—original draft; formal analysis; writing—review and editing. **Harry Tattan‐Birch:** Conceptualization; writing—review and editing; formal analysis. **Melissa Oldham:** Conceptualization; writing—review and editing. **Katherine East:** Conceptualization; writing—review and editing. **Hannah Walsh:** Conceptualization; writing—review and editing. **Sarah Jackson:** Conceptualization; writing—review and editing; funding acquisition; supervision; methodology.

## DECLARATION OF INTERESTS

M.O. has received funding from Alcohol Change UK (ACUK) for a separate ongoing research project on alcohol‐free and low‐alcohol drinks that began in January 2025. Since beginning that project, the authors have become aware that ACUK received <0.6% of its funds in 2024–2025 from Lucky Saint, an organisation that produces and sells non‐alcoholic drinks and owns a pub that sells standard alcoholic drinks. In March 2025, Lucky Saint became an associate member of The Portman Group, a self‐regulatory organisation that is fully funded by the alcohol industry. All other authors have no conflicts of interest to declare.

## Supporting information


**Figure S1:** Prevalence of tobacco use by ethnicity; pooled 2013–2025.
**Figure S2**. Geometric mean cigarettes per day among those who smoke cigarettes: pooled 2013–2025.
**Figure S3a.** Geometric mean units per week by ethnicity: pooled 2014–2025.
**Figure S3b.** Geometric mean EWAC score by ethnicity: pooled 2014–2025.
**Figure S4.** Prevalence of risky drinking (AUDIT‐C ≥ 5) among people who do or do not smoke by ethnicity: pooled 2014–2025.
**Table S1.** Drinking among adults from Gypsy or Traveller communities compared with other ethnic groups; January 2014‐Febuary 2025.

## Data Availability

Data are available on reasonable request. Please see https://smokinginengland.info/resources/sts-documents for information on applying for data access.
