# Supplementary material for: Smoking and drinking among the Gypsy and Traveller communities: A population study in England
Source: Addiction. 2026 Feb 17;121(5):1128–39. doi: 10.1111/add.70330 (PMC13088939; doi:10.1111/add.70330)
Supplement: Supplementary file 1 — Figure S1: Prevalence of tobacco use by ethnicity; pooled 2013–2025. Figure S2. Geometric mean cigarettes per day among those who smoke cigarettes: pooled 2013–2025. Figure S3a. Geometric mean units per week by ethnicity: pooled 2014–2025. Figure S3b. Geometric mean EWAC score by ethnicity: pooled 2014–2025. Figure S4. Prevalence of risky drinking (AUDIT‐C ≥ 5) among people who do or do not smoke by ethnicity: pooled 2014–2025. Table S1. Drinking among adults from Gypsy or Traveller communities compared with other ethnic groups; January 2014‐Febuary 2025. [file ADD-121-1128-s001.docx]

*Other includes those who reported they were another ethnicity, don’t know, or refused

*Other includes those who reported they were another ethnicity, don’t know, or refused

*Other includes those who reported they were another ethnicity, don’t know, or refused

AUDIT-C = Alcohol Use Disorder Identification Test – Consumption

EWAC = Estimator of Weekly Alcohol Consumption scale

| Table S1: Drinking among adults from Gypsy or Traveller communities compared with other ethnic groups; January 2014-Febuary 2025 | | | | | | | | | |
| --- | --- | --- | --- | --- | --- | --- | --- | --- | --- |
|  | All participants | |  | Men | |  | Women | | |
| **Units per week** (n=144,487)^a^ | AGM (95%CI) | AGMR (95%CI) | Sig | AGM (95%CI) | AGMR (95%CI) | Sig | AGM (95%CI) | AGMR (95%CI) | Sig |
| Gypsy or Traveller | 3.5(2.5-4.9) | 1 | Ref | 4.7(2.9-7.6) | 1 | Ref | 2.6(1.7-4.1) | 1 | Ref |
| Other White | 3.4 (3.4-3.5) | 0.98 (0.70-1.36) | .883 | 4.6(4.5-4.6) | 0.97(0.61-1.56) | .909 | 2.5(2.5-2.5) | 0.97(0.62-1.53) | .894 |
| Mixed/ multiple ethnicity | 2.7 (2.5-2.8) | 0.76 (0.54-1.06) | .109 | 3.3(3.1-3.6) | 0.70(0.44-1.14) | .153 | 2.1(1.9-2.3) | 0.80(0.51-1.27) | .351 |
| Asian/British Asian | 1.6 (1.6-1.7) | 0.47 (0.33-0.66) | **<.001** | 2.1(2.0-2.3) | 0.45(0.28-0.72) | **.001** | 1.3(1.2-1.4) | 0.50(0.31-0.79) | **.003** |
| Black/Black British | 1.4 (1.4-1.5) | 0.40 (0.29-0.56) | **<.001** | 1.7(1.6-1.8) | 0.36(0.22-0.58) | **<.001** | 1.1(1.1-1.2) | 0.44(0.28-0.69) | **<.001** |
| Arab | 1.9 (1.5-2.4) | 0.54 (0.36-0.82) | **.004** | 2.5(1.9-3.4) | 0.53(0.30-0.94) | **.029** | 1.5(1.0-2.1) | 0.56(0.31-1.03) | .061 |
| Other ^b^ | 2.2 (2.1-2.4) | 0.63 (0.45-0.89) | **.008** | 3.0(2.7-3.3) | 0.63(0.39-1.02) | .063 | 1.6(1.5-1.8) | 0.62(0.39-0.99) | **.045** |
| **EWAC score** (n=144,018)^b^ |  |  |  |  |  |  |  |  |  |
| Gypsy or Traveller | 8.2(7.0-9.5) | 1 | Ref | 9.2(7.5-11.3) | 1 | Ref | 7.3(5.9-9.0) | 1 | Ref |
| Other White | 7.6(7.6-7.6) | 0.93(0.80-1.08) | .326 | 8.8(8.7-8.8) | 0.96(0.78-1.18) | .668 | 6.5(6.5-6.5) | 0.89(0.72-1.09) | .257 |
| Mixed/ multiple ethnicity | 6.7(6.6-6.9) | 0.83(0.71-0.96) | **.013** | 7.5(7.3-7.8) | 0.82(0.67-1.01) | .068 | 6.0(5.8-6.2) | 0.81(0.66-1.01) | .058 |
| Asian/British Asian | 5.4(5.3-5.5) | 0.66(0.57-0.77) | **<.001** | 6.2(6.0-6.3) | 0.67(0.54-0.83) | **<.001** | 4.7(4.6-4.9) | 0.65(0.52-0.80) | **<.001** |
| Black/Black British | 5.2(5.0-5.2) | 0.62(0.54-0.73) | **<.001** | 5.6(5.5-5.8) | 0.61(0.50-0.76) | **<.001** | 4.6(4.5-4.7) | 0.62(0.50-0.77) | **<.001** |
| Arab | 5.7(5.1-6.3) | 0.69(0.58-0.83) | **<.001** | 6.3(5.5-7.2) | 0.69(0.54-0.88) | **.003** | 5.3(4.4-6.4) | 0.72(0.54-0.95) | **.021** |
| Other ^b^ | 6.1(5.9-6.3) | 0.74(0.64-0.87) | **<.001** | 7.1(6.8-7.4) | 0.77(0.63-0.96) | **.018** | 5.1(4.9-5.3) | 0.70(0.56-0.87) | **.001** |
| a Excludes those who did not currently drink (n=59,195), or responded don’t know or refused (n=2,928) to Audit 1 or 2 questions.  b Excludes those who did not currently drink (n=59,195), or responded don’t know or refused (n=3,397) to Audit 1, 2 or 3 questions.  b ‘Other’ includes those who reported they were another ethnicity, don’t know, or refused  Analysis are adjusted for age, gender, survey year and the onset of the COVID-19 pandemic  EWAC= Estimator of Weekly Alcohol Consumption scale  AGM= Adjusted geometric mean  AGMR= Adjusted geometric mean ratio  Sig= Statistical significance  Bold denotes a p value of <.05. | | | | | | | | | |

*Other includes those who reported they were another ethnicity, don’t know, or refused

AUDIT-C = Alcohol Use Disorder Identification Test – Consumption
